# Utility of Radiofrequency Echographic Multi-spectrometry in Evaluating Bone Health in Patients With Spondylarthritis

**DOI:** 10.7759/cureus.84069

**Published:** 2025-05-13

**Authors:** Ionut-Andrei Badea, Mihai Bojinca, Violeta Bojinca, Mihaela Milicescu, Andreea-Ruxandra Ilina, Madalina-Stefania Vulcan, Stefan-Sorin Arama

**Affiliations:** 1 Internal Medicine and Rheumatology, "Dr. I. Cantacuzino" Clinical Hospital, Carol Davila University of Medicine and Pharmacy, Bucharest, ROU; 2 Rheumatology and Internal Medicine, “Sfanta Maria” Clinical Hospital, Carol Davila University of Medicine and Pharmacy, Bucharest, ROU; 3 Internal Medicine, Colentina Clinical Hospital, Bucharest, ROU; 4 Internal Medicine and Rheumatology, Colentina Clinical Hospital, Bucharest, ROU; 5 Physiopathology, Carol Davila University of Medicine and Pharmacy, Bucharest, ROU

**Keywords:** bone mineral density, dual x-ray absorptiometry, fragility fracture, radiofrequency echographic multispectrometry, spondylarthritis

## Abstract

Introduction: Osteoporosis, a condition characterized by reduced bone strength and increased fracture risk, frequently coexists with spondyloarthritis (SpA), an inflammatory rheumatic disease. Dual-energy X-ray absorptiometry (DXA), the gold standard for diagnosing osteoporosis, faces limitations in SpA patients due to spinal deformities and calcifications. Some studies have shown overestimation of BMD values, especially in individuals with syndesmophytic bridges and coexistent mechanical modifications such as osteophytes and discopathic lesions.

Materials and methods: A cross-sectional study was performed to compare the results of bone mineral density (BMD) lumbar spine evaluations performed with Radiofrequency Echographic Multi-spectrometry (REMS) between a control group and a group of patients recently diagnosed with axial spondyloarthritis (axSpA) who did not follow any specific treatment. All study participants were informed about the objectives, examinations performed, and the use of anonymous data in databases to conduct statistical analyses and publish findings in reference medical scientific journals.

Results: The ratio between male and female participants differed between the study groups. No significant differences were observed between the control and SpA groups, in both female and male subjects, with regard to BMD values. When factoring in the age of the patients, it was observed that the mean age of the control group was 55.53 years, while that of the SpA group was 36.96 years. This suggests that the BMD values of younger SpA patients were equivalent to those of older individuals in the control group.

Conclusion: REMS is a useful tool for evaluating BMD both in the general population and in SpA patients. Furthermore, the lack of any statistically significant correlation between the control and SpA groups confirms that the rheumatic disease influences lumbar bone mineralization. Also, similar BMD values were observed between the groups, despite significantly different mean ages, supporting the conclusion that bone degradation appears early in the evolution of SpA and can be correctly identified using REMS.

## Introduction

Radiofrequency Echographic Multi-spectrometry (REMS) technology has the potential to accurately determine bone mineral density (BMD) and also provide an indicator of bone fragility through the Fragility Score (FS) [1]. The applicability of the method in spondyloarthritis (SpA) is not yet fully studied, with only a limited number of studies available in the scientific literature. Only one study could be identified suggesting that the BMD values obtained by REMS at the femoral level are equivalent to those obtained by DXA, but with differences noted in the assessment of the lumbar spine [2].

Axial spondyloarthritis (axSpA) is frequently associated, both in the literature and in clinical practice, with abnormalities of bone mineralization and its complications, such as fragility fractures. These abnormalities may also be present prior to the clinical onset of the disease, raising the potential for identifying bone fragility complications at the time of axSpA diagnosis [3]. Some authors have also drawn attention to the falsely increased BMD results obtained by DXA in patients with axSpA and radiological structural lesions in the lumbar spine [4]. In this context, we aimed to investigate in depth the data related to the REMS method, which has been reported to automatically remove artifacts that can lead to overestimation of BMD values in patients with SpA (https://www.echolightmedical.com/nasa/, accessed on May 12, 2025).

The usefulness of REMS and FS in the context of rheumatic inflammatory pathologies has also begun to be studied. The applicability of REMS in determining BMD in RA has been demonstrated in several reference studies, particularly in comparison with DXA, showing good sensitivity and specificity in diagnosing bone mineralization abnormalities [5,6]. In terms of costs, some authors estimate a significant reduction related to the use of REMS, due to faster diagnosis and, naturally, an increased treatment rate for the prevention of fragility fractures [7]. In SpA, REMS has the potential to provide a complex and complete assessment of bone density and fragility, with the ability to filter out artifacts related to secondary structural changes in the lumbar spine (e.g., syndesmophytes, calcifications), thereby compensating for the overestimation of BMD noted in several DXA-based assessments [8]. The main objective of this study is to compare, identify, and characterize the differences between DXA and REMS examinations in the lumbar spine in patients diagnosed with axSpA. A secondary aim is to identify the subgroup of patients who would benefit most from lumbar spine REMS examination for diagnosis and prognosis.

## Materials and methods

A cross-sectional study was designed to compare a significant control group from the general population and a group of patients diagnosed with axial spondyloarthritis (axSpA), based on the Assessment of SpondyloArthritis (ASAS) classification criteria for axial spondyloarthritis, approved by the European League Against Rheumatism (EULAR) and the American College of Rheumatology (ACR) [9]. These patients were identified and recruited during their periodic hospital or clinical routine visits. Thus, 535 participants were included in the control group, among the usual patients who arrived at the hospital for routine investigations, and 76 individuals diagnosed relatively recently with axSpA (maximum of two years' disease duration), and who had not received any disease-modifying antirheumatic drug (DMARD) treatment. This difference between the study group sizes is notable, making statistical analysis more complex and requiring ANOVA testing and Bonferroni post hoc analysis to provide an adequate statistical assessment, with the risk of obtaining false-negative 95% CI values. The enrollment period of the two groups took place between May 2020 and August 2023 for the control group and between July 2022 and June 2024 for the axSpA group, in a state hospital and in two private clinics. The REMS examination was performed at the state hospital and in a private center by trained physicians in a standardized manner, involving examination of the lumbar spine and obtaining BMD values. The examinations were free of charge, with no additional costs allocated to patients. All study participants were informed about the objectives, examinations performed, and the use of anonymous data in databases to perform statistical analyses and publish findings in reference medical scientific journals.

To ensure correct inclusion of the SpA group, a number of inclusion criteria were formulated for individuals with axSpA: clear diagnosis confirmed by axSpA based on ASAS-EULAR classification criteria [9]; absence, prior to and at the time of examination, of any DMARD with the potential to influence bone metabolism; DXA examination (maximum three months prior to the REMS examination) or scheduled for DXA examination based on clinical recommendations within a maximum of three months after the REMS examination; the DXA examination must cover the lumbar spine (L1-L4 vertebrae) and the examination of at least one femur; existence of a lumbar spine radiograph, at least in latero-lateral (profile) incidence, performed within a maximum of 12 months prior to REMS. The exclusion criteria formulated for this study include: coexistence of comorbidities that can influence bone metabolism and mineralization (e.g., Cushing's syndrome, endocrine pathologies, cortisone treatment less than three months after the REMS examination or chronic cortisone treatment, use of antidepressant treatments from any pharmaceutical class); lack of recommendations for DXA examination; patients undergoing classic synthetic, biological, or targeted synthetic DMARD therapy; lack of lumbar spine imaging; refusal to perform or medical contraindications for performing magnetic resonance imaging (MRI) examinations.

After verifying the inclusion and exclusion criteria, demographic data were gathered. Weight and height measurements were performed in order to calculate the body mass index (BMI) and classify participants as underweight, normal weight, overweight, or obese. Medical documents were evaluated to identify comorbidities and concomitant medications. Laboratory data obtained during normal patient visits to the hospital or clinic were checked. The X-rays of axSpA patients and, where applicable, their MRI investigations were evaluated and stored electronically, while preserving confidentiality. After examination of the documents, REMS investigation was performed, and BMD values were electronically stored. Statistical analysis was performed with Minitab v.20 (Minitab LLC) and online on DataTab (datatab.net, DATAtab: Online Statistics Calculator, DATAtab e.U., Graz, Austria).

## Results

In the control group, other comorbidities were identified, such as arterial hypertension (n = 387, 72.33%), hyperlipidemia (n = 392, 73.27%), type 2 diabetes mellitus (n = 37, 6.91%), autoimmune thyroiditis with normal thyroid function without any treatment (n = 42, 7.85%), and β-thalassemia minor (n = 2, 0.37%). In the SpA group, three patients were identified with hyperlipidemia (3.94%) and two patients with arterial hypertension (2.63%). Descriptive analysis shows differences between the two study groups with regard to gender, with males comprising the majority of SpA patients, while the control group had a significant female population, probably because postmenopausal women have a clear recommendation for at least one baseline DXA evaluation (Table 1). Another aspect that can be observed is the statistical difference between DXA and REMS measurements when comparing controls and SpA patients, whereas REMS differentiation between the two groups did not have the same statistical strength (p = 0.118). The mean disease duration for SpA patients was 8.4 months (two-sample t-test comparing males and females, p < 0.001).

**Table 1:** Descriptive statistics comparing the two study groups. ^a^χ² = 0.68, p = 0.409; ^b^Independent-samples t-test, p < 0.001. ^c^Mann–Whitney U test, p = 0.002. ^d^Mann–Whitney U test, p = 0.09. SpA, spondyloarthritis; DXA, dual-energy X-ray absorptiometry; BMD, bone mineral density; REMS, radiofrequency echographic multi-spectrometry.

Parameter	Control (n = 535)	SpA Patients (n = 76)	p-value
Male/Female^a^	99/436	59/17	0.409
Age mean (range) years^b^	55.53(20–91)	36.96(20–65)	<0.001
Underweight (%)	9(1.68%)	0(0%)	-
Normal weight (%)	143(26.73%)	15(19.74%)	-
Overweight (%)	357(66.73%)	41(53.95%)	-
Grade I obesity (%)	26(4.86%)	20(26.32%)	-
Lumbar DXA BMD mean (StDev)^c^	0.75 ± 0.11	0.79 ± 0.05	0.002
Lumbar REMS BMD mean (StDev)^d^	0.7 ± 0.1	0.71 ± 0.08	0.09
Vitamin D mild deficit (percentage of individuals)^b^	27.11%	23.07%	<0.001

**Figure 1:**
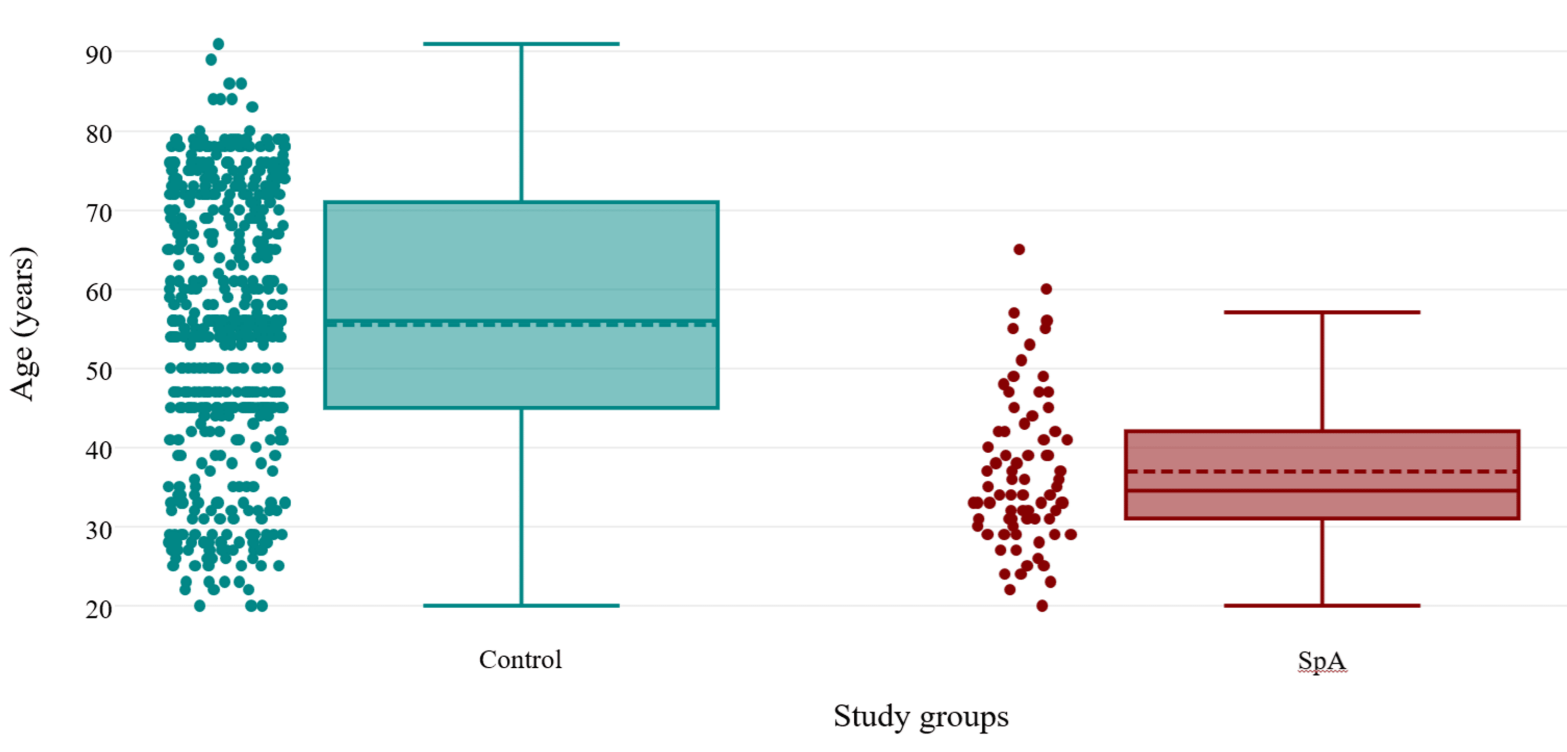
The age distribution of the two study groups (independent sample t-test, p-values<0.001). Levene test of equality of variance yielded a p<0.001, confirming that there are no equal variances between the study groups. SpA, Spondyloarthritis.

Further, when comparing the groups by age, we observed another important difference. The mean age of the control group was 55.53, while the mean age for the SpA group was 36.96 (Figure 1, p < 0.001). 

**Figure 2:**
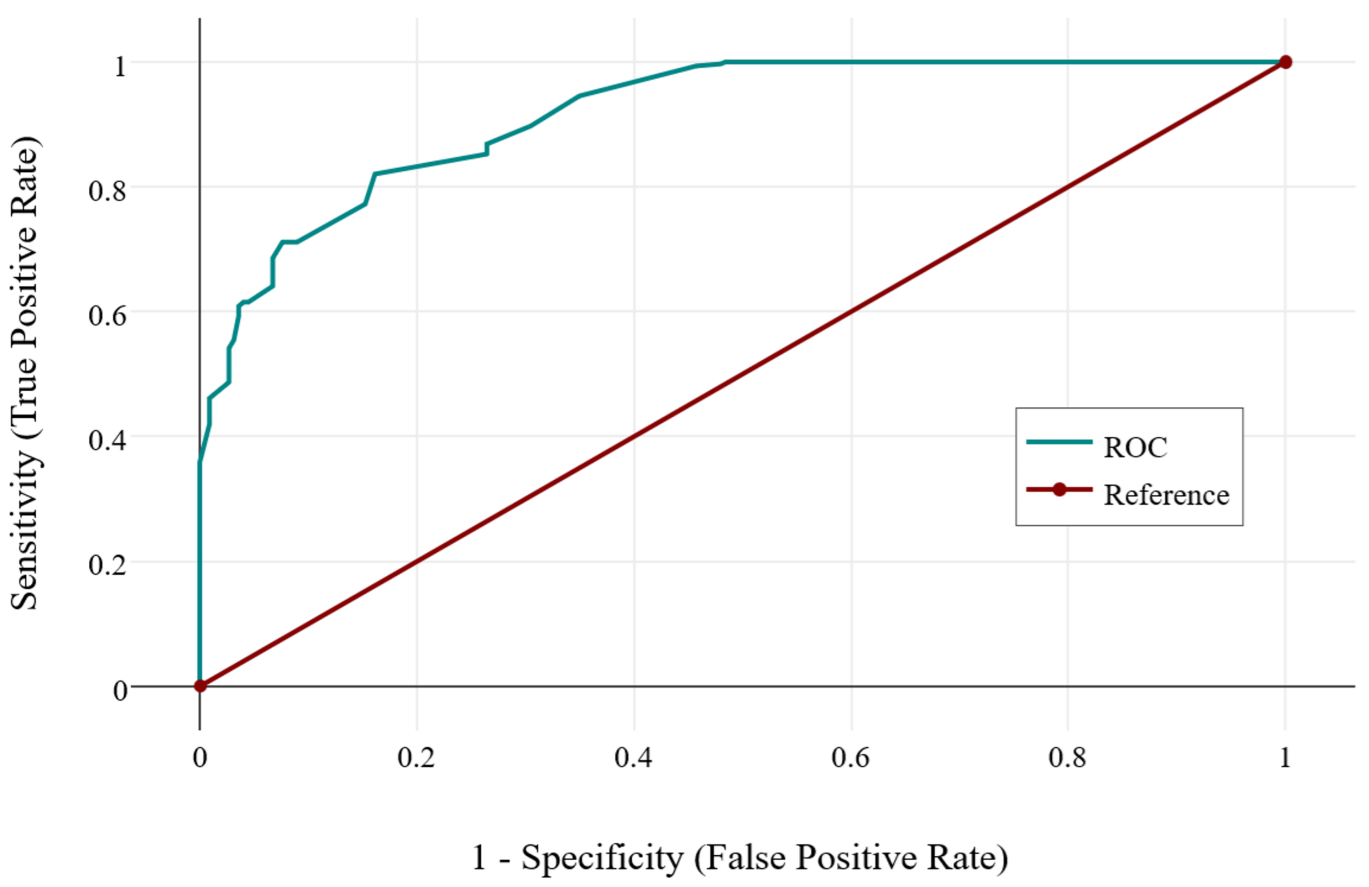
ROC curve describing the relationship between age and vertebral structural modifications found on imagistics in the control group. Chi^2 ^= 347.98, p < 0.001. ROC, receiver operating characteristic.

**Figure 3:**
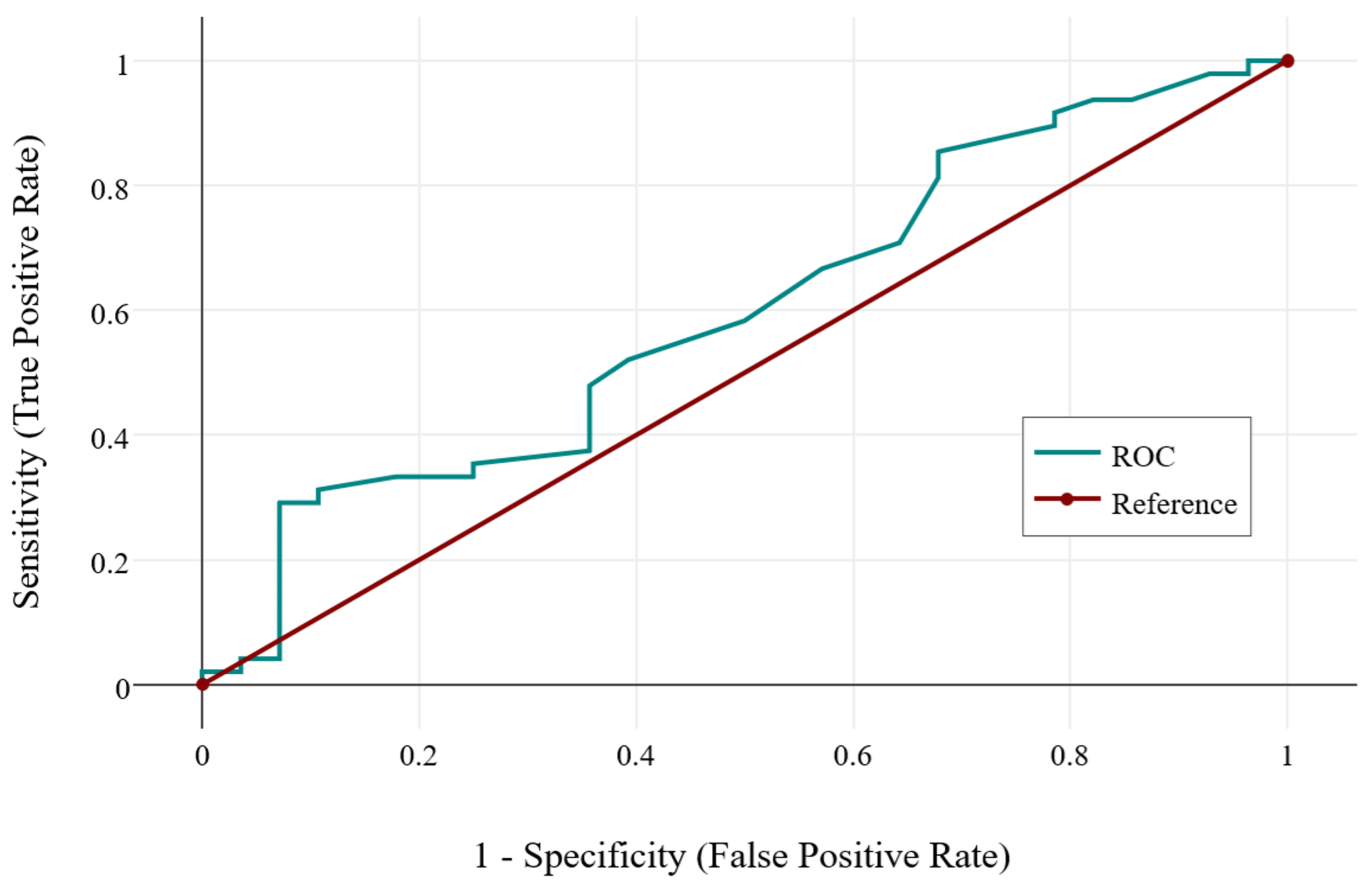
ROC curve describing the relationship between age and vertebral structural modifications found on imagistics in the SpA group. Chi^2 ^= 2.35, p = 0.125. ROC, receiver operating characteristic; SpA, spondyloarthritis.

Correlation between DXA and REMS measurements of BMD in the lumbar region yielded a statistically insignificant, negligible correlation in the control group (r = 0.07, p = 0.119), and a statistically significant, moderate, negative correlation in the SpA group (r = -0.32, p = 0.005). This analysis suggests that the two methods calculate different BMD values in both study groups. This can stem from different causes: either one or both of the measurements were not correctly performed, REMS is not a reliable method, or there are artifacts that can perturb BMD calculation in both methods. After reviewing the imaging data from the two groups, we found that older patients in the control group had marked structural modifications of the spine (e.g., osteophytes, scoliosis, vertebral hemangiomas, and vertebral fractures). Since vertebral modifications usually appear with age, a logistic regression was performed for both study groups. In controls, age is the main factor determining structural damage (Figure 2, Chi² = 347.98, p < 0.001). On the other hand, in the SpA group, age is not related to structural modification, but rather these appear in the context of the disease itself (Figure 3, Chi² = 2.35, p = 0.125).

Mixed model ANOVA testing in the control group showed differences between DXA and REMS BMD measurements (F(1, 533) = 77.08, p < 0.001). Structural modifications appeared to have influenced the measurement (F(1, 533) = 30.01, p < 0.001), and the differences in measurement may be related to these specific modifications (F(1, 533) = 30.01, p < 0.001). The same cannot be said about the SpA group, where the difference in BMD measurement is statistically significant between the two methods (F(1, 74) = 38.11, p < 0.001), but this does not appear to stem from the presence of structural modifications (e.g., syndesmophytes, vertebral squaring, etc.; F(1, 74) = 0.16, p = 0.69), with neither method being influenced by them (F(1, 74) = 0.16, p = 0.85).

**Figure 4:**
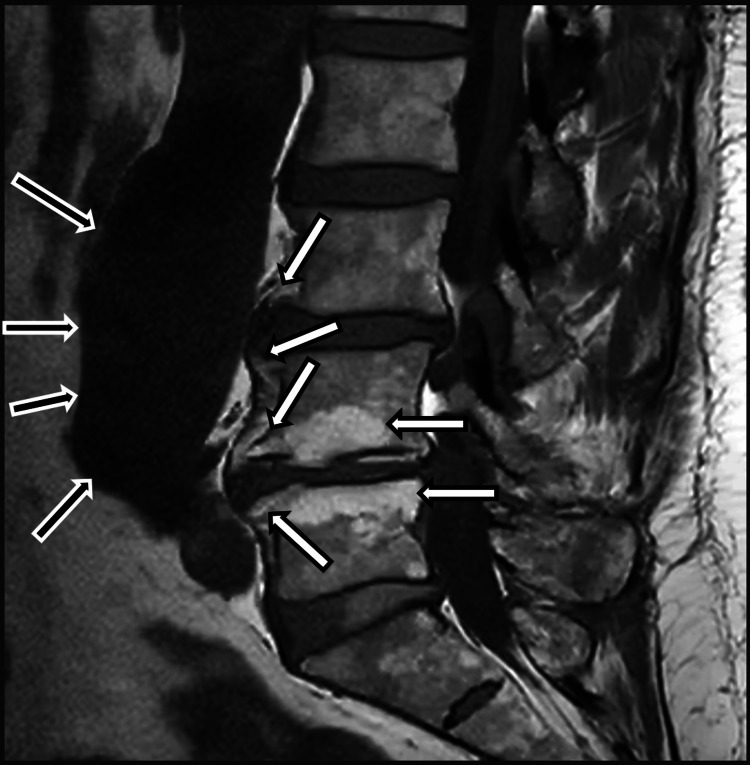
MRI T1 acquisition of the lumbar spine from subject "iv-377". The white arrows point out multiple vertebral modifications such as osteophytes and fatty infiltration of the vertebral plateaus. The black arrows identify an aneurysmal dilation of the abdominal aorta, associating parietal thrombosis. MRI, magnetic resonance imaging.

**Figure 5:**
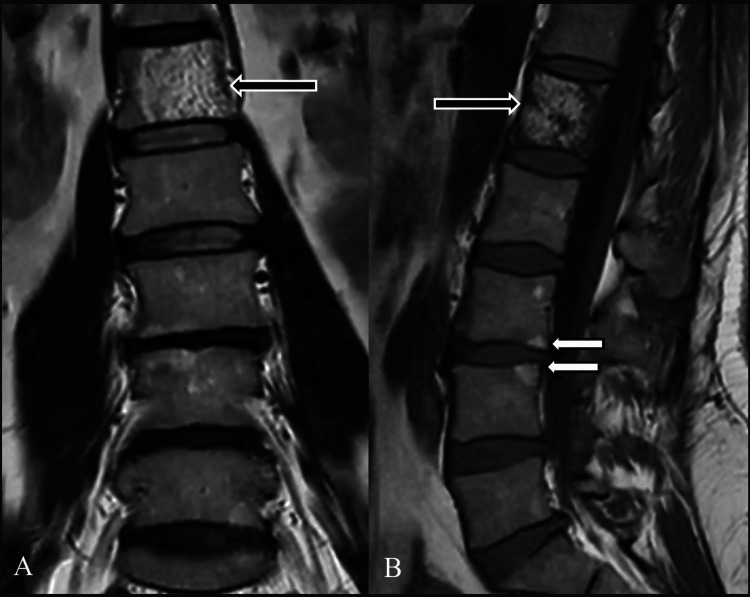
MRI T1 acquisitions in frontal plane (A) and lateral plane (B) of the lumbar spine in a patient diagnosed with SpA. The white arrows point at two vertebral corner fatty infiltration modifications, while the black arrows point at the presence of a large hemangioma occupying approximately 90% of the L1 vertebral body. MRI, magnetic resonance imaging; SpA, spondyloarthritis.

Examples of modifications identified during examination can be seen in Figure 4 (from the control group) and Figure 5 (from the SpA group).

Since spinal modifications generally appeared in older patients, we divided the control group into a group of patients under the age of 45 (inclusive) and a group of patients above this age. When performing the Kruskal-Wallis test to compare REMS BMD results, we found that the SpA measurements were significantly different from those of controls aged above 45 years (Table 2). Factoring in the mean age of the SpA group, 36.96 years, we can consider that, in these patients, no obvious differences were found compared to the corresponding age group in the general population (Chi² = 40.84, df = 2, p < 0.001).

**Table 2:** Dunn-Bonferroni-Tests comparing REMS measurements in control groups and SpA group. REMS, radiofrequency echographic multi-spectrometry; BMD, bone mineral density; SpA, spondyloarthritis.

Comparison	Test Statistic	Std. Error	Std. Test Statistic	p-value	Adj. p-value
REMS lumbar BMD (control<45 years) - REMS lumbar BMD (control>45 years)	102.33	16.61	6.16	<0.001>	<0.001>
REMS lumbar BMD (control<45 years) - REMS lumbar BMD (SpA)	34.61	24.54	1.41	0.158	0.475
REMS lumbar BMD (control>45 years) - REMS lumbar BMD (SpA)	-67.72	22.22	-3.05	0.002	0.007

Dunn-Bonferroni comparisons suggest that the BMD values of the younger SpA group are statistically comparable to those of the younger (under 45 years old) control group, which could indicate that there is no noticeable demineralization in SpA.

## Discussion

The statistically significant differences between the two methods suggest that both offer different results regarding lumbar BMD measurements, especially in the general population. These differences can stem from the presence of vertebral structural modifications. It is worth mentioning that the presence of bone structural changes in the lumbar spine may represent the only factors influencing the results of BMD, although several authors in the scientific literature have raised the suspicion that structural changes may overestimate the results of DXA [10]. The relatively weak correlation between DXA and REMS in determining BMD may indicate that either method can be influenced by the aforementioned structural factors (osteophytes, syndesmophytes, aortic calcifications, hemangiomas, etc.). We must also take into account that the scientific literature has identified weak correlations between DXA examination and textural analysis by computed tomography (CT) [11]. A similar study comparing REMS and CT results regarding bone mineralization might prove extremely useful.

Either DXA is a sufficiently accurate method for detecting bone density in all patient groups (controls and SpA), while REMS yields inaccurate results, or the reverse may be true. Potentially, the same artifacts identified during the study could influence BMD measurements differently in DXA and REMS, respectively. Slart et al. draw attention to the importance of correct positioning and measurement technique for DXA, suggesting that many erroneous results may stem from incorrect examination or the presence of artifacts, such as osteophytes, voluminous vertebral hemangiomas, DISH, and abdominal aortic changes [12]. Whatever the underlying issue, REMS requires further studies on larger populations and comparison with highly sensitive diagnostic procedures and bone biomarkers to fully understand its utility in SpA patients. The main advantage of REMS, from a medical standpoint, is that it is a non-irradiating procedure, making it suitable for use in SpA patients under the age of 65, the age at which DXA testing is typically recommended [13]. It must also be considered in future scientific endeavors that population dynamics are constantly evolving. In recent years, especially in European populations but also internationally, reduced sun exposure and vitamin D deficiency have become increasingly prevalent in studied populations, with significant consequences for patients with rheumatic disorders [14].

The relative superiority of REMS compared to DXA, as discussed in the literature, cannot yet be confirmed based on the results of statistical analysis [15]. A large number of well-known studies and case reports have highlighted the limitations of DXA scanning, with similar cases in the literature reporting BMD misinterpretation and ultimately relying on clinician experience to determine the optimal management. FRAX scores can be used to assess vertebral fragility and may serve as a useful tool in offering a more complete picture [16], as they do not always require BMD values [17]. Corroborating the results of this study with those of others using a similar design, suggesting that REMS may overcome the overestimations associated with DXA [18], supports the view that REMS may provide a complementary and possibly more reliable method for measuring spinal BMD in both the general population and in SpA patients, although additional large-scale studies with balanced groups are needed.

## Conclusions

The present study provides useful information on the applicability of REMS both in the general population and in patients newly diagnosed with axSpA. The uncertain correlation between the two methods in the axSpA and control groups needs to be further explored by comparing REMS with highly accurate densitometric evaluations, such as quantitative CT. The advantages of REMS compared to DXA in this context are related to the speed of the method and its non-irradiating nature, with the potential for evaluating pregnant women with axSpA. The overall cost and accessibility of REMS and DXA must also be taken into consideration.
